# Observations on the Efficacy of Two Methods for the Treatment of Upper Eyelid Retraction in Thyroid-Associated Ophthalmopathy

**DOI:** 10.1155/2021/9514279

**Published:** 2021-03-18

**Authors:** Dongping Li, Fengyuan Sun

**Affiliations:** ^1^Department of Oculoplastic Surgery, Hankou Aier Eye Hospital, Wuhan 430000, China; ^2^Eye Institute and School of Optometry, Tianjin Medical University Eye Hospital, Tianjin 300384, China; ^3^Tianjin International Joint Research and Development Centre of Ophthalmology and Vision Science, Tianjin 300384, China

## Abstract

**Objective:**

To observe the clinical efficacy of periocular injection of triamcinolone acetonide (TA) and subpalpebral injection of botulinum toxin type A (BTXA) for the treatment of thyroid-associated ophthalmopathy (TAO) with mild unilateral upper eyelid retraction.

**Method:**

This was a prospective randomized controlled study. A total of 68 cases of stable thyroid-associated ophthalmopathy with mild upper eyelid retraction were collected at Hankou Aier Eye Hospital from Jan. 2015 to Dec. 2018 and randomly divided into two groups. Group A contained 33 patients who were administered TA by periocular injection once every 3 weeks for a total of 3 times. Group B contained 35 patients who were given a single subpalpebral administration of BTXA. The efficacy in the two groups was observed.

**Results:**

Compared with the two groups, the effective rate in both groups was 100% at 1 week and 1 month after treatment. The effective rate of Group A remained 100% at 3 months after treatment, and that of Group B decreased to 88.6%. At 1 week after treatment, the degree of correction in Group B was greater than that in Group A (*p* < 0.001). At 1 month after treatment, it was not significantly different between the two groups (*p* > >>0.05). At 3 months after treatment, it was less in Group B than in Group A (*p* < 0.001). In Group A, there was one case of transient amaurosis, two cases of periorbital hemorrhage and swelling, and one mild case of sunken eyes. In Group B, four cases experienced recurrence after 3 months.

**Conclusion:**

Periocular injection of TA and subpalpebral injection of BTXA offer definite therapeutic efficacy for mild upper eyelid retraction associated with thyroid disease. The former has a long treatment period, large procedural risks, and stable efficacy. The latter is a simple procedure with a short treatment period but can easily recur.

## 1. Introduction

The initial symptom in 90% of TAO cases is upper eyelid retraction [1, 2], a symptom which causes patients to appear as if glaring straightforward. Unilateral cases can exhibit pseudoeyelid retraction caused by contralateral ptosis, which causes an obvious asymmetry of the eyes [3]. Serious cases may be unable to close the eyelids completely (lagophthalmos) and suffer from surface eye pathologies as a result [4]. In the case of patients with mild eyelid retraction, glucocorticoids and BTXA are the principal drugs used for nonsurgical treatment. Periocular injection of TA is commonly used to control ocular symptoms in active TAO [5], but recent literature rarely focuses on upper eyelid retraction [6]. BTXA's specific chemical denervation effect on muscle tissue makes it particularly suitable for diseases related to the eye muscles [7, 8]. Gallo et al. [9] reported the use of BTXA for the treatment of esotropia in children. Wei et al. [10] used percutaneous injection of BTXA into the levator palpebrae superioris muscle to treat different degrees of TAO-associated upper eyelid retraction and achieved good results. This study selected two groups of TAO patients with mild eyelid retraction, using either periocular injection of TA or subpalpebral injection of BTXA into levator palpebrae superioris, also achieving good results.

## 2. Materials and Methods

### 2.1. General Information

We collected TAO patients from the eyelid orbital department of Hankou Aier Eye Hospital from January 2015 to December 2018. The inclusion criteria are as follows: (1) the clinical diagnosis of all patients was TAO, with mild unilateral upper eyelid retraction of 1~2 mm. (2) Thyroid function in all patients was within normal range. (3) Clinical activity score (CAS) ≤3. (4) They had no obvious exophthalmos in the affected eye (measurement discrepancy <2 mm). (5) Their intraocular pressure was within normal range. (6) None of the patients had contraindications to triamcinolone acetonide or BTXA. The exclusion criteria are as follows: (1) all patients had no history of active eyelid inflammation, eyelid trauma, and operation of levator palpebral complex. (2) Pregnant and lactating women were excluded. (3) Patients with random blood glucose ≥10 mmol/L were excluded if they were complicated with diabetes.

Patients were randomly divided into two groups. Patients of Group A were administered TA via periocular injection once every 3 weeks for a treatment cycle of 3 injections. Patients of Group B were given a single administration of BTXA via subpalpebral injection, which counted as one treatment cycle. All injections were administered by the same experienced attending physician. This study was approved by our ethics committee and informed consent was given by patients.

### 2.2. Dispensing Method

- *Preparation of TA.* We shake the TA solution well and extract 20 mg (0.5 ml) with a syringe, then add 2% lidocaine 0.2 ml
- *Preparation of BTXA.* BOTOX (Allergan), refrigerated at -20°C~-5°C, was taken out and placed at room temperature for 10 minutes, then diluted to 40 U/ml with 2.5 ml normal saline. Use immediately after dilution or refrigerate at 2°C~8°C and use within 4 hours

### 2.3. Injection Methods

**Figure 1:**
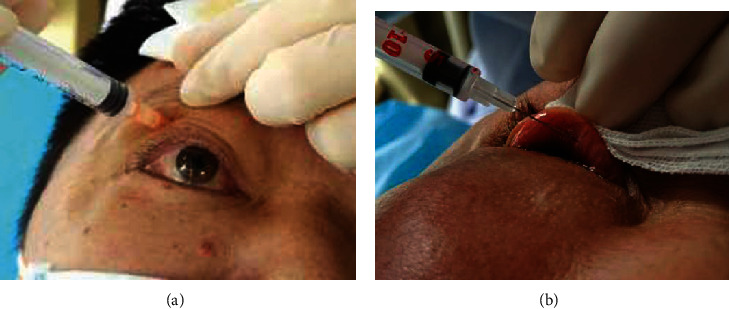
(a) Injection of TA. (b) Injection of BTXA. At 1 week, 1 month, and 3 months after completion of a treatment cycle, we observed the two groups for the effective rate of the treatment, the degree of eyelid correction, and the occurrence of complications.

- *TA Injection.* We direct the patient to stare at a target below the nose, locate the orbital margin, and insert the needle close to the supraorbital margin between its central and lateral thirds along the orbital wall toward the equatorial region, to a depth of about 25 mm. Then, we instruct the patient to rotate the eyeball to confirm that the needle is not touching. Retract the plunger to check for blood reflux, and if none, slowly inject the liquid into the orbit. No obvious resistance should be felt on injection. Gently withdraw the needle, apply sterile gauze to the eyelid, and gently press the eyeball and orbit for 5 minutes. We instruct the patient to blink several times and look at the target. Their vision should be as before, with no obvious bleeding or eye discomfort (Figure 1(a))
- *BTXA Injection.* A special insulin syringe with a 30G needle was used to extract the appropriate amount of liquid preparation. The affected eye was anesthetized with 0.4% obucaine hydrochloride, and the upper eyelid was retroverted using an eyelid retractor to expose a certain width and length of the fornix conjunctiva. Avoiding blood vessels, inject the preparation into the levator palpebrae superioris at the medial and lateral borders of the central third of the tarsal plate. Dosage: 1.5 U per point (Figure 1(b))

### 2.4. Measurement Standards and Efficacy Criteria

- *Measurement Standards.* Only cases of unilateral eyelid retraction were accepted in this study. The difference between the heights of the palpebral fissures of the affected and healthy eyes was taken as the initial degree of retraction, and the degree of correction was calculated on the basis of this measurement and defined as the distance of inferior displacement of the upper eyelid margin after treatment. The patient was asked to sit still and look straight ahead; then, the palpebral fissure was measured 3 times with a ruler, and the mean value was recorded
- *Efficacy Criteria.* Accounting for human error, a change in eyelid retraction greater than or equal to 0.5 mm was defined as effective and less than 0.5 mm as ineffective

### 2.5. Statistical Methods

We used the SPSS 22.0 statistical software to analyze the data. The effective rates of treatment in the two groups were compared using Fisher's exact test, and the degree of eyelid correction was compared using the two independent samples *t* test, with *a* = 0.05 as the testing standard.

## 3. Results

### 3.1. General Patient Information Results

A total of 68 patients were collected in this study, including 31 males and 37 females, aged 22 to 59 years, with eyelid retraction of 1 to 2 mm. There was no significant difference in age, eyelid retraction, CAS score, intraocular pressure (IOP), and thyroid function index (FT3, FT4, TSH) between the two groups (all *p* > 0.05), which was comparable. The basic information of the patients was shown in Table 1.

### 3.2. Treatment Effective Rate

The symptom of upper eyelid retraction remained significantly improved in both groups 1 week and 1 month after the completion of one treatment cycle, an effective rate of 100%. Three months after treatment, the effective rate of Group A was still 100%, and Group B was 88.6% (Table 2). The difference between the two groups was not statistically significant. On follow-up, patients in Group A reported that their upper eyelid felt more relaxed than before on opening the eye. One week after treatment, patients in Group B complained of a sensation of heaviness in the affected eyelid, which disappeared after one month.

### 3.3. Correction of Eyelid Retraction

**Figure 2:**
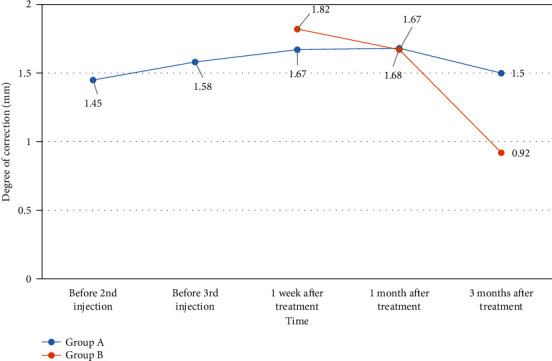
Degree of correction after injection in both groups. Note: Group A: periocular injection of TA; Group B: subpalpebral injection of BTXA.

Measurements of eyelid retraction correction at 1 week, 1 month, and 3 months after treatment in both groups are presented in Table 3. One week after treatment, the degree of correction in Group B was greater than in Group A, and the difference was statistically significant (*p* < 0.001). One month after treatment, there was no significant difference in the degrees of correction between the two groups (*p* > 0.05). Three months after injection, the degree of correction in Group B was less than in Group A, and the difference was statistically significant (*p* < 0.001).  Figure 2 shows that the degree of correction in Group A was more stable at 3 months, while Group B showed a downward trend in therapeutic effect at 3 months.

### 3.4. Complications

One patient in Group A developed transient amaurosis after the 3rd injection and then improved on his own. There were two patients with slight periorbital bleeding and swelling, which disappeared on their own with no effect on visual acuity. One patient developed slightly sunken eyes and periorbital fat atrophy. Four patients in Group B experienced a recurrence of upper eyelid retraction at 3 months after injection and requested another injection. No other significant complications were noted.

## 4. Discussion

As the most common and earliest symptom of TAO, upper eyelid retraction is a frequent cause of outpatient visits. For mild upper eyelid retraction, although the procedure has undergone continuous improvement and progress made in the techniques used [11, 12], the amount of operation is still difficult to quantify accurately, and the difficulty of treatment is still due to the undercorrection and recurrence after operation. Moreover, due to the traumatic nature of surgery and the fearfulness of patients, it is not the first choice for treatment.

Drug therapy is usually the preferred option for patients with such concerns. Lee et al. [13] and Xu et al. [14] were able to use glucocorticoid therapy to effectively alleviate the symptoms of upper eyelid atrophy and swelling using subconjunctival injection of TA. However, Cao Yujin et al. [15] observed two cases of subconjunctival deposition after subconjunctival injection of TA. According to Li Yan et al. [16], 8% of patients exhibited patchy subconjunctival drug deposition after using the fornix conjunctiva method. As a result, the authors believe that because TA is a suspension with a relatively large volume of injection, and since subconjunctival injections are prone to deposition, uneven dispersion of the solution results. The drug diffuses easily because the orbital space is large and the tissue loose, which is consistent with the view of Kozaki [17]. Therefore, for this study, a periocular route of administration was utilized. As for treatment with BTXA, Wei et al. [10] injected the levator palpebrae muscle percutaneously, 25 mm along the orbital apex through the central orbital rim. Although deep injection into the belly of the levator palpebrae superioris would be more effective [18], the target site is not directly visible, and there are no signs for assessing whether the injection was successful, while also posing a risk of ocular injury and bleeding. The levator palpebrae has a clear projection onto the conjunctival surface and can be directly exposed. Therefore, we adopted subpalpebral injection under direct vision for this study. Zeng et al. [19] and Nava et al. [20] also used this route of administration.

In this study, both drugs showed good therapeutic effects, but results varied in different patients, mainly reflected in the following three points: (1) the principles of the two treatments are different. Wang et al. [21] proposed that TAO patients showed enlargement of the superior rectus/levator palpebrae superioris complex, with an average CAS score of 2.1, suggesting that levator palpebrae superioris and superior rectus may be the first muscle tissues to develop lesions in the non-active stage. Byun et al. [22] measured the volume of the levator palpebrae complex using imaging methods and also found that upper eyelid retraction was associated with a volume increase. Both BTXA and TA aim to treat the upper eyelid by weakening the levator palpebrae superioris muscle. The former blocks the release of acetylcholine at the neuromuscular junction and causes a temporary decline in its function after local injection, thus providing a symptomatic treatment for upper eyelid retraction [10]. The latter is widely used in the treatment of autoimmune diseases to inhibit the immune system, anti-inflammatory reactions, and its other pharmacological effects, offering an etiological treatment [6, 18]. As a synthetic long-acting glucocorticoid, TA has a sustained action time and strong effect, making periocular injection a more appropriate route of administration for avoiding systemic effects. The results of this study show that patients in the TA treatment group had a sense of relief after injection, and Xu et al. [14] reached similar conclusions when evaluating the therapeutic effect of TA using MRI signal intensity. However, patients in the BTXA treatment group felt a sensation of heaviness after injection. The reason appears related to its mechanism of action and target. The main target tissue of TA periocular injection is soft tissue, including the levator palpebrae superioris muscle, exerting an anti-inflammatory effect not only on the muscle itself but also on the orbital soft tissue and other extraocular muscles to a certain extent. As a result, immune inflammation is reduced in periorbital tissue and the patient feels no discomfort on opening the eye. This also explains the case of periorbital fat atrophy after TA injection in this study [23]. However, the target tissue of BTXA injection is precisely the levator palpebrae superioris, with only a small diffusion area around the injection site and no involvement of orbital soft tissue. Moreover, the levator palpebrae has a strong affinity for BTXA. Locally targeted injection of BTXA weakens the levator palpebrae, resulting in a lack of strength to open the eye and a short-term increase in the sensation of heaviness [10]. (2) Patients have different experiences of the treatment. In this study, the treatment cycle of TA therapy was 9 weeks, but some researchers have used a once-weekly injection schedule [16, 19]. In light of the metabolic profile of TA, we used a schedule of 3 periocular injections, one every 3 weeks. BTXA only requires a single local injection per treatment cycle, which is more advantageous for patient follow-up compliance. Glucocorticoids have an array of side effects, such as raising intraocular pressure, affecting the gastrointestinal tract, and raising blood pressure and blood sugar, which increase the psychological burden of patients [5, 6, 13]. Although BTXA poses risk of ptosis, diplopia, recurrence, and so on, these side effects are reversible as the drug is metabolized, so in terms of safety, this drug is more in line with the psychological expectations of patients. In terms of the injection method, TA therapy is administered via periocular injection, which is a deep injection site that is not directly visible and poses a certain risk of orbital hemorrhage [17]. BTXA can be injected into a shallow, visible site, and avoids vascular injection, so from a procedural risk point of view, BTXA is more acceptable to patients. It can be seen that compared with TA therapy, the treatment with BTXA was a better experience that was more acceptable to the patients. (3) Treatment results differ. Data recorded on follow-up shows that each TA treatment showed progressive improvement over the last, achieving a certain cumulative effect, which is an innovation finding of this study. Effects were still relatively obvious and stable 3 months after treatment. Possible reasons include that only stable patients with mild eyelid retraction were selected for this study, or that the effects of long-acting glucocorticoids on target tissue are able to bring immune inflammation under better control, and not less likely to rebound [13]. However, BTXA treatment reached its best efficacy at 1 month after treatment, and although it still had some therapeutic effect at 3 months, it had significantly diminished and patients had recurrences that required repeat injections. This is consistent with the findings of previous studies [10]. In terms of stability of therapeutic effect, TA therapy is obviously superior to BTXA therapy. It should be noted that patients who experienced recurrences in this study were all patients with a relatively large degree of eyelid retraction initially. Possible reasons include the following: (a) all patients received a fixed dosage, which May have been insufficient for some patients with slightly greater eyelid retraction. The dosage should be increased as appropriate. (b) This study included only patients with stable upper eyelid retraction, and some patients with a tendency to mild fibrosis may have a poor drug response. (c) Some patients may have an innate ability to metabolize BTXA at a higher rate, shortening the duration of efficacy.

Lee suggests that the position of the upper eyelid is significantly associated with exophthalmos, CAS score, and thyroid hormone immunoglobulin [24]. All patients involved in this study were mild patients with stable disease in the early stages, and their exophthalmos values were within the normal range. Their condition was stable throughout the treatment, and no significant active manifestations were seen. The stability of the treatment effect depends largely on control of the primary disease; therefore, endocrinological maintenance of thyroid function is also a key component of treatment [2].

In summary, both periocular injection of TA and subpalpebral injection of BTXA demonstrated efficacy in the treatment of TAO-associated mild upper eyelid retraction. The former has a stable treatment effect, but a long treatment period and relatively high procedural risk. The latter has a high recurrence rate, but the procedure is simple, the treatment period is short, and the injection can be repeated. Therefore, patients can be offered different nonsurgical options for treatment of TAO-associated mild upper eyelid retraction based on the acceptability of the risks to the patient, treatment compliance, and so on. This study has some limitations: the sample size of this study is relatively small, and the current study results can only draw a preliminary conclusion. In the later stage, the sample size should be expanded to conduct detailed grouping studies, in order to explore the dose-effect relationship.

## Figures and Tables

**Table 1:** Comparison of general information between the two groups.

Groups	Age (years)	Eyelid retraction	CAS score	IOP	TSH	FT3	FT4
Group A	35.48 ± 5.63	1.60 ± 0.21	1.39 ± 0.83	16.85 ± 2.07	2.16 ± 0.92	5.39 ± 0.99	16.11 ± 2.23
Group B	35.71 ± 5.23	1.58 ± 0.18	1.23 ± 0.81	15.89 ± 2.13	2.02 ± 0.87	5.27 ± 0.94	15.85 ± 1.97
*t*	0.174	0.364	-0.834	-1.894	-0.641	-0.511	-0.509
*p*	0.862	0.717	0.407	0.063	0.524	0.611	0.612

Note: Group A: periocular injection of TA; Group B: subpalpebral injection of BTXA.

**Table 2:** Effective rate in both groups.

Group	1 week after treatment			1 month after treatment			3 months after treatment		
	Effective	Ineffective	Effective rate (%)	Effective	Ineffective	Effective rate (%)	Effective	Ineffective	Effective rate (%)
Group A	33	0	100	33	0	100	33	0	100
Group B	35	0	100	35	0	100	31	4	88.6
*p*			1			1			0.115

Note: Group A: periocular injection of TA; Group B: subpalpebral injection of BTXA.

**Table 3:** Degree of correction after treatment (mm, mean ± SD).

Group	Before 2nd injection	Before 3rd injection	1 week after treatment	1 month after treatment	3 months after treatment
Group A	1.45 ± 0.14	1.58 ± 0.13	1.67 ± 0.15	1.68 ± 0.15	1.50 ± 0.14
Group B			1.82 ± 0.15	1.67 ± 0.12	0.92 ± 0.28
*t*			-4.09	0.41	10.62
*p*			0.001	0.685	0.001

Note: Group A: periocular injection of TA; Group B: subpalpebral injection of BTXA.

## Data Availability

The data used to support the findings of this study are available from the corresponding author upon request.
